# Use of a High-Purity Factor X Concentrate in Turkish Subjects with Hereditary Factor X Deficiency: Post Hoc Cohort Subanalysis of a Phase 3 Study

**DOI:** 10.4274/tjh.2017.0446

**Published:** 2018-05-25

**Authors:** Ahmet F. Öner, Tiraje Celkan, Çetin Timur, Miranda Norton, Kaan Kavaklı

**Affiliations:** 1 Yüzüncü Yıl University Faculty of Medicine, Department of Pediatric Hematology, Van, Turkey; 2 İstanbul University Cerrahpaşa Faculty of Medicine, Department of Pediatric Hematology and Oncology, İstanbul, Turkey; 3 İstanbul Medeniyet University, Göztepe Training and Research Hospital, Clinic of Pediatric Hematology, İstanbul, Turkey; 4 Bio Products Laboratory Ltd., Elstree, Hertfordshire, United Kingdom; 5 Ege University Faculty of Medicine, Department of Pediatric Hematology, İzmir, Turkey

**Keywords:** Clinical trial, Clotting factor concentrate, Efficacy, Factor X deficiency, Orphan drug, Safety

## Abstract

Hereditary factor X (FX) deficiency is a rare bleeding disorder more prevalent in countries with high rates of consanguineous marriage. In a prospective, open-label, multicenter phase 3 study, 25 IU/kg plasma-derived factor X (pdFX) was administered as on-demand treatment or short-term prophylaxis for 6 months to 2 years. In Turkish subjects (n=6), 60.7% of bleeds were minor. A mean of 1.03 infusions were used to treat each bleed, and mean total dose per bleed was 25.38 IU/kg. Turkish subjects rated pdFX efficacy as excellent or good for all 84 assessable bleeds; investigators judged overall pdFX efficacy to be excellent or good for all subjects. Turkish subjects had 51 adverse events; 96% with known severity were mild/moderate, and 1 (infusion-site pain) was possibly pdFX-related. These results demonstrate that 25 IU/kg pdFX is safe and effective in this Turkish cohort (ClinicalTrials.gov identifier: NCT00930176).

## Abstract (tr)

Kalıtsal faktör X (FX) eksikliği, akraba evliliklerinin yüksek oranda görüldüğü ülkelerde daha sık olan nadir bir kanama bozukluğudur. Prospektif, açık etiketli, çok merkezli bir faz 3 çalışmada 6 ay ila 2 yıl boyunca gerektiği zaman veya kısa dönemli profilaktik olarak 25 IU/kg plazma kaynaklı FX (pdFX) uygulanmıştır. Türk hastalarda (n=6) kanamaların %60,7’si hafiftir. Her kanamayı tedavi etmek için ortalama 1,03 infüzyon gerekmiş ve kanama başına ortalama toplam doz 25,38 IU/kg olmuştur. Türk hastalar değerlendirilebilir 84 kanamanın tümü için pdFX etkililiğini mükemmel veya iyi olarak derecelendirmiştir; araştırmacılar genel pdFX etkililiğinin tüm hastalarda mükemmel veya iyi olduğu kararına varmıştır. Türk hastalarda 51 advers olay gözlenmiştir; şiddeti bilinenlerin %96’sı hafif/orta derecededir ve 1’i (infüzyon bölgesi ağrısı) muhtemelen pdFX ile ilişkili olmuştur. Bu sonuçlar 25 IU/kg pdFX kullanımının bu Türk kohortunda güvenli ve etkili olduğunu ortaya koymaktadır (ClinicalTrials.gov tanımlayıcısı: NCT00930176).

## Introduction

Hereditary factor X (FX) deficiency (FXD) is a rare, autosomal recessive coagulation disorder most prevalent in countries with high rates of consanguineous marriage [1,2,3,4,5,6,7,8]. Patients with severe FXD commonly present with bleeding into joints, muscles, or mucous membranes [1,3]. Hereditary FXD is often treated with fresh-frozen plasma (FFP) or prothrombin complex concentrates (PCCs) [9,10], but single-factor concentrates, when available, are recommended for treatment of rare bleeding disorders [11].

A high-purity, high-potency, plasma-derived FX concentrate (pdFX; Bio Products Laboratory Ltd., Elstree, UK) is approved in the USA and the EU for on-demand treatment and bleeding episode control in subjects aged ≥12 years with hereditary FXD [12]. pdFX efficacy and safety were demonstrated in 5 subjects with hereditary FXD undergoing surgery [13] and in 16 subjects with hereditary FXD in a phase 3 trial conducted in the USA, the UK, Spain, Germany, and Turkey [14].

This analysis evaluated pdFX use in the Turkish cohort (a homogeneous subgroup in terms of the *F10* mutation) from the phase 3 trial [14].

## Materials and Methods

This was a post hoc analysis of 6 Turkish subjects enrolled in a prospective, open-label, multicenter, nonrandomized phase 3 study (ClinicalTrials.gov identifier, NCT00930176; EudraCT identifier, 2009 0111145-18) [14] with independent ethics committee approval for each study center, conducted in accordance with good clinical practice guidelines [15]. All subjects provided written informed consent.

As reported previously [14], enrolled subjects were aged ≥12 years with moderate or severe hereditary FXD (FX activity [FX:C] <5 IU/dL) with ≥1 spontaneous/menorrhagic bleed in the previous 12 months treated with FFP, PCCs, or a factor IX/X concentrate. Subjects received on-demand pdFX at 25 IU/kg for 6 months to 2 years until ≥1 bleed had been treated; pdFX was also used as short-term preventative therapy and presurgical prophylaxis [13].

### Assessments

pdFX efficacy, pharmacokinetics (PK), and safety were assessed for the Turkish cohort as previously described for the overall cohort [14,16]; optional *F10* genotyping was also performed [17]. Subjects evaluated treatment efficacy for each bleed, and investigators evaluated treatment efficacy for each subject. Bleeds were categorized as menorrhagic, covert, or overt, and pdFX efficacy for each bleed was categorized as “excellent,” “good,” “poor,” or “unassessable” [14]. An independent data review committee evaluated each bleed for assessability and severity.

PK assessments were performed at baseline and 6 months or after ≥1 bleed had been treated with pdFX as described previously [16]. Plasma FX:C levels were measured via a one-stage clotting assay, and incremental recovery and half-life were calculated.

Safety and tolerability assessments included adverse events (AEs), infusion-site reactions, thrombogenicity markers, and viral serology. FX inhibitor development was analyzed using activated partial thromboplastin time-based inhibitor screens and the Nijmegen-Bethesda assay.

## Results

The Turkish cohort (Table 1) had a history of severe bleeds treated using FFP or PCCs; one subject (17%) and 3 subjects (50%, including the only subject with moderate FXD) had received >150 days of exposure to FFP and PCCs, respectively. All 6 subjects had the same homozygous missense mutation in the *F10 *gene (p.Gly262Asp), including 3 who were known relatives.

### Hemostatic Efficacy

**Figure 1:**
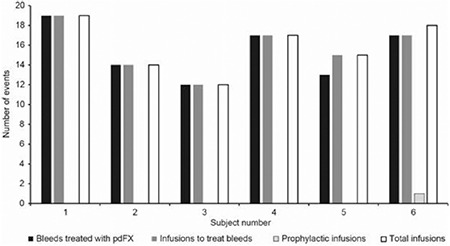
Summary of bleeding episodes treated with plasma-derived FX (Turkish cohort). pdFX: plasma-derived FX.

Of 92 pdFX-treated bleeds (range, 12-19; Figure 1), 84 were eligible for primary efficacy analysis (Table 2). The median number of bleeds was 1.05 per subject per month overall (range, 0.8-1.2), and 1.1 bleeds per month for the subject with moderate FXD. The majority of bleeds (60.7%) were minor. Major bleeds (39.3% of all episodes) included spontaneous bleeding, injury, and menorrhagia.

A total of 95 pdFX infusions (94 exposure days) were administered (mean total dose, 22,596 IU or 389 IU/kg) to treat a bleed (n=94) or for short-term preventative use (n=1) (Table 3). A mean of 1.03 infusions were used to treat each bleed, and mean total dose per bleed was 25.38 IU/kg. All 6 Turkish subjects completed the study and then received on-demand pdFX compassionate use for 1 year. During this time, 1 subject experienced a subdural hematoma successfully treated with pdFX, followed by weekly pdFX prophylaxis (2000 IU; ~30.8 IU/kg).

Subject-rated efficacy was “excellent” or “good” for each of the 84 pdFX-treated assessable bleeds. Investigators rated pdFX efficacy (on-demand, preventative, or surgical) as “excellent” in 4 subjects (67%) and “good” for 2 subjects (33%).

FX:C PK parameters following single intravenous pdFX doses did not differ significantly between baseline and repeat PK assessment visits. Mean pdFX incremental recovery was slightly lower in the Turkish cohort than the overall cohort (1.77 vs. 2.00 IU/dL per IU/kg, respectively), while the mean terminal half-life was similar (29.7 vs. 29.4 h, respectively).

### Safety and Tolerability

Of 51 AEs reported by the Turkish subjects, 44 of 46 (96%) with known severity were mild or moderate. The most frequently reported AE was upper respiratory tract infection (9 events in 4 subjects, none of which were considered by the investigators to be related to pdFX). Of the 51 AEs, 1 event in 1 subject (mild infusion-site pain) was considered possibly pdFX-related; no AEs were considered probably or very likely pdFX-related, and no AEs resulted in death.

There were no inhibitors to FX, viral seroconversions, or hypersensitivity reactions to pdFX. No evidence of thrombotic events or clinical signs of thrombogenicity were observed.

During the year of compassionate use, no product-related AEs were reported. One pdFX infusion was given to treat bleeding due to a urinary tract infection during pregnancy, with no adverse effect on the baby.

## Discussion

This post hoc analysis demonstrated the efficacy, PK, and safety of pdFX in Turkish subjects with moderate or severe hereditary FXD. One subject with moderate FXD (FX:C 1 IU/dL) nonetheless had severe bleeding diathesis based on his bleeding and treatment history.

The Turkish cohort required fewer infusions to treat each bleed than the overall study cohort [14] (mean, 1.03 vs. 1.21 doses) and consequently a lower total dose per bleed (mean, 25.38 vs. 31.00 IU/kg). The percentage of minor bleeds was higher in the Turkish cohort than in the overall study population (60.7% vs. 47.1%), and preventative use was much lower (mean, 0.06 vs. 1.64 infusions per month). The slightly lower mean pdFX incremental recovery among Turkish subjects versus the overall study population [16] may derive from the small sample size. Across 94 exposure days, only 1 AE in 1 subject was considered by the investigators to be possibly treatment-related.

All Turkish subjects had a homozygous *F10* mutation (p.Gly262Asp) resulting in an identical amino acid substitution. A recent study of 12 Turkish patients with severe FXD identified p.Gly262Asp in 11 of 12 patients (92%), this mutation being associated with severe bleeding symptoms, suggesting the potential value of mutational screening analysis in Turkey and certain areas of Iran [18]. Other regional studies have also suggested a correlation between genotype and clinical manifestations of hereditary FXD [9,19]; additional studies are needed, however, to confirm these findings.

## Conclusion

In conclusion, pdFX is the first highly purified FX concentrate developed for patients with hereditary FXD. The treatment success rate observed in Turkish subjects (100%) was comparable with that in the overall study population (98.4%) [14]. As hereditary FXD is a rare disorder, this post hoc analysis is limited by a small sample size. Nevertheless, these results demonstrate that 25 IU/kg pdFX was safe and effective in Turkish patients with moderate or severe hereditary FXD for on-demand treatment of bleeding episodes.

## Figures and Tables

**Table 1:**
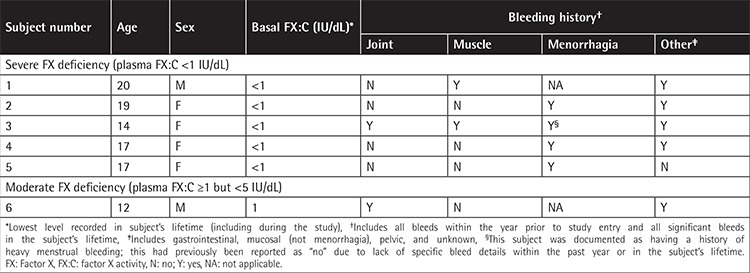
Subjects’ demographics and clinical characteristics (Turkish cohort; n=6).

**Table 2:**
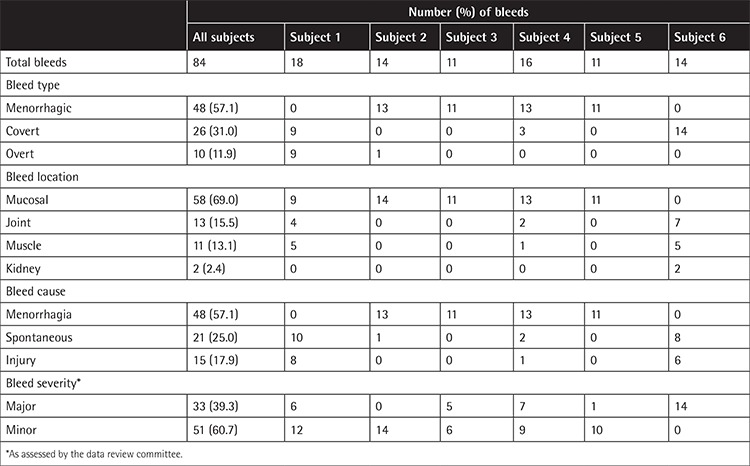
Characteristics of assessable* bleeding episodes (n=84) treated with plasma-derived FX and analyzed (Turkish cohort).

**Table 3:**
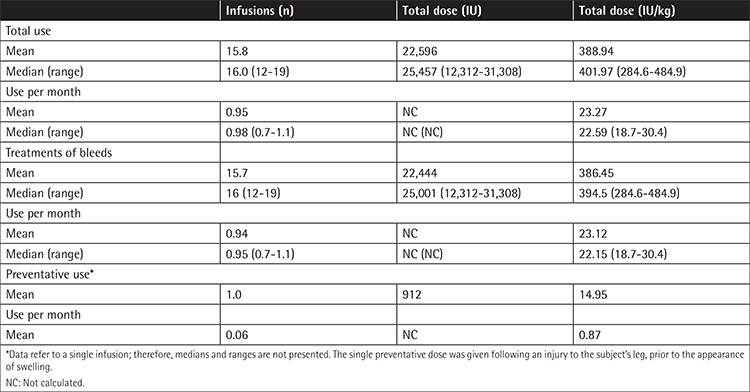
Summary of plasma-derived FX infusions (Turkish cohort).
